# 2021 revised algorithm for the management of knee osteoarthritis—the Chinese viewpoint

**DOI:** 10.1007/s40520-021-01906-y

**Published:** 2021-06-29

**Authors:** Zhiyi Zhang, Cibo Huang, Yongping Cao, Rong Mu, Mun Chan Zhang, Dan Xing, Dongwei Fan, Yunpong Ding, Junhuan Guo, Yong Hou, Lin Jianhao, Nicola Veronese, Jean-Yves Reginster, Olivier Bruyere, Etienne Cavalier, Huaihuan Zhang

**Affiliations:** 1 Department of Rheumatology, The First Affiliated Hospital of Harbin Medical University, Harbin, China; 2 Department of Rheumatology, Beijing Hospital, Beijing, China; 3 Department of Orthopedics, Peking University First Hospital, Beijing, People’s Republic of China; 4 Arthritis Clinic and Research Center, Peking University People’s Hospital, Peking University, Beijing, 100044 China; 5 Department of Orthopedics, Peking University Third Hospital, Beijing, China; 6 Department of Orthopedics, The Affiliated Hospital of Academy of Military Medical Sciences, PLA 307Th Hospital, Beijing, 100071 China; 7 Department of Rheumatology and Clinical Immunology, Peking Union Medical College Hospital, Chinese Academy of Medical Sciences, Peking Union Medical College, National Clinical Research Center for Dermatologic and Immunologic Diseases, Beijing, 100730 China; 8 Department of Internal Medicine and Geriatrics, Geriatric Unit, University of Palermo, Palermo, Italy; 9 WHO Collaborating Centre for Public Health Aspects of Musculoskeletal Health and Aging, Liège, Belgium; 10 Department of Public Health, Epidemiology and Health Economics, University of Liège, CHU Sart Tilman B23, 4000 Liège, Belgium; 11 Department of Clinical Chemistry, University of Liège, CHU de Liège, 4000 Liège, Belgium; 12 Department of Science and Technology, China Electric Power Research Institute, Beijing, China

**Keywords:** Knee osteoartrhitis, Patented crystalline glucosamine sulfate, Symptomatic slow-acting drugs for osteoarthritis, Algorithm, China

## Abstract

**Aim:**

The European Society for Clinical and Economic Aspects of Osteoporosis, Osteoarthritis and Musculoskeletal Diseases (ESCEO) algorithm for the management of knee osteoarthritis (OA) is available worldwide from 2014, but in 2019 an update was published. Based on this algorithm, a Working Group (WG), including ESCEO members and Chinese experts, wished to see how the new ESCEO algorithm was perceived by Chinese experts in knee OA and how it was integrated into their clinical practice.

**Methods:**

A WG was held between members of the international ESCEO task force and a group of Chinese experts.

**Results:**

Non-pharmacological approach should be combined with pharmacological interventions. In step 1, symptomatic slow-acting drugs for osteoarthritis (SYSADOA) are the most important background drugs. Evidence, supported by high-quality research, is available only for crystalline glucosamine sulfate (pCGS) and chondroitin sulfate. Topical NSAIDs could be used as an additional option. In step 2, oral NSAIDs could be useful, but cardiovascular/renal/gastrointestinal profiles of the patients should be considered. Intra-articular hyaluronic acid and corticosteroids are alternative to oral NSAIDs, but the evidence is still limited. If steps 1 and 2 are not sufficient, weak opioids could be used**.** Overall, the conclusions of the ESCEO algorithm are accepted in China for products available in this country. The WG suggests the importance of economic studies, specifically made in China.

**Conclusion:**

This work provides evidence-based advice to establish a treatment algorithm in knee OA, for practical implementation in clinical practice in China.

## Introduction

Osteoarthritis (OA) is common, being characterized by typical signs with relevant consequences on functional decline, finally resulting in a relevant loss in quality of life [1, 2]. Knee is the most common localization of the knee [3]. Symptomatic forms seem to affect more than 250 million people worldwide [3]. Knee OA is estimated among the most common causes of global disability in terms of Disability-Adjusted Life Years (DALY) [4–6].

The European Society for Clinical and Economic Aspects of Osteoporosis, Osteoarthritis and Musculoskeletal Diseases (ESCEO) published some indications for the management of knee OA in 2014, creating a treatment algorithm that gives practical guidance for interventions and therefore guiding physicians through steps based on the severity of knee OA [7]. Since the publication of the 2014 algorithm, new papers are available. In particular, the safety of the medications used for knee OA is increasing in importance [8–12].

In 2019, a new ESCEO algorithm [13] was published taking into account the recent research on efficacy and safety of medications commonly used for knee OA and the GRADE (Grading of Recommendations Assessment, Development and Evaluation) evaluation was added, to better highlight the evidence used in the algorithm [14]. In these last five years, the ESCEO algorithm has been diffused worldwide and endorsed by many national societies including China, Russia and Southeast Asia [15–19]. Recently, Southeast Asian and European ESCEO experts have produced a new guidance providing evidence-based and easy-to-follow advices on how to establish a treatment algorithm in knee OA, for practical implementation in clinical practice in Southeast countries [20].

Given this background, a working group (WG) was formed including members of the international ESCEO task force (N.V., O.B. E.C., and J.-Y.R.) and a group of Chinese experts in knee OA (working group, WG) to see how key opinion leaders in China perceive ESCEO algorithm and how it can be combined, with their own clinical daily practice, for harmonizing and optimizing the management of their patients affected by knee OA.

## Epidemiological data of knee osteoarthritis in China

The interest of Chinese public health authorities and physicians in knee OA is exponentially increasing as well as the epidemiological interest in the potential risk factors for knee OA. Knee OA is a very common condition in China, with about 8% of people with a symptomatic form [21] and with a prevalence of mild, moderate and severe knee OA of 1.5%, 3.3%, and 3.9%, respectively [22]. The risk factors for knee OA have a critical importance in research regarding knee OA epidemiology. Among them, obesity seems to be one of the most important [23]. Some recent data, specific for the Chinese population, indicated that the actual prevalence of obesity in this country is continuously increasing: obesity in China is a major health concern according to the World Health Organization, with a prevalence of about 5%, but greater than 20% in some cities where fast food is popular [24]. Obesity is strongly associated with the presence of knee OA, as a large meta-analysis reported [25]. Another factor that seems to be important in Chinese vision of knee OA is the presence of abnormal joints at birth (i.e. congenital abnormalities) and genetic factors [26]. These people, in fact, were born with abnormally formed joints, vulnerable to mechanical wear, causing early degeneration and loss of joint cartilage. Moreover, some genetic factors, specific for Chinese people, seem to be associated with an increased risk of knee OA [27]. Finally, another important risk factor in China for knee OA is the prevalence of metabolic abnormalities, in particular, type 2 diabetes mellitus [28, 29].

## Non-pharmacological treatment in the 2019 ESCEO algorithm

Non-pharmacological interventions (information/education; weight loss in case of overweight or obesity; physical exercise mixing aerobic and strengthening exercises) have a relevant role being supported by a high level of evidence according to the GRADE [7, 13, 30]. However, it is widely accepted that the real effect of these interventions is limited and their feasibility in the long term is still unclear [31]. In China, the core interventions, including exercise, education, and weight loss, are widely used. Furthermore, the Chinese experts, during the workshop, highlighted the importance of Tai-Chi [32, 33] and acupuncture [34, 35], typical Chinese traditions, for their patients.

## Pharmacological treatment in the 2019 ESCEO algorithm

### Step 1: background treatment

#### Paracetamol

Paracetamol (acetaminophen) is among the most used medication in the world, including the treatment of knee OA symptoms. In 2014 ESCEO reported that paracetamol has only a marginal effect on pain without any significant effect on stiffness or physical function in knee OA [36–38]. In the last years, several concerns are rising regarding the safety of paracetamol, due to the increasing research reporting that the risk of gastrointestinal (GI), cardiovascular (CV), hepatic and renal adverse events (AEs) [39] and finally mortality [40] should be deeply considered. Based on this evidence (robust evidence of AEs and limited effect), the 2019 ESCEO algorithm recommends that paracetamol should be used only as rescue medication, in case of the inefficacy of the background therapy [13]. Moreover, as observed by the Chinese experts of the WG, paracetamol is not widely used in China and, when used in more severe forms of knee OA.

#### SYSADOAs

In 2014 and 2019 versions of the ESCEO algorithm, Step 1 treatment recommends to start background therapy with long-term SYSADOAs (Symptomatic Slow-acting Drugs for Osteoarthritis) [7, 13]. However, this class includes several products such as glucosamine, chondroitin, diacerein, and avocado soybean unsaponifiables (ASU), which are supported by varying degrees of clinical efficacy and safety data.

Glucosamine and chondroitin are natural compounds. Glucosamine hydrochloride (GHCl) is obtained using an extraction process and often used as a nutraceutical or over-the-counter (OTC) products. Conversely, glucosamine sulfate (GS) is a more complex product, obtained only by a proprietary semi-synthetic route and stabilization process. This important process is used only in the prescription-grade crystalline glucosamine sulfate (pCGS) [41]. Finally, some recent observational findings in the UK Biobank suggest that GS can be useful in decreasing cardiovascular risk [42]. Unfortunately, multiple formulations of GS are available [43], both as prescription-grade products and OTC, but the latter products usually have limited amounts of glucosamine. On the contrary, only pCGS is able to deliver in an appropriate way a clinical efficacious glucosamine bioavailability and plasma concentration, resulting in good clinical efficacy [44–51]. On the contrary, GHCl and non-crystalline glucosamine sulfate products (usually consisting of GHCl with the addition of sodium sulfate to get a “sulfate” labelling) are ineffective in the treatment of knee OA [44, 46, 52–56]. A similar evidence can be applied to chondroitin sulfate [57–63], even if this product in the world (including China) is less commercially diffused. Based on the scientific evidence available, ESCEO specifically recommends the use of pCGS and long-acting chondroitin sulfate products (the latter of which are not available in China) in both versions of the algorithm published in 2014 and 2019 [7, 13].

The judgement regarding SYSADOAs is also based on their safety since, except for diacerein, several randomized placebo-controlled trials (RCTs) have demonstrated that SYSADOAs are safe, both considering total and specific events [11]. When discussing of pCGS and CS, we have a robust literature indicating that the use of pCGS is able to reduce by about 50% the indication for the TKR and the use of medications showed in step 2 in a similar way [64, 65]. Moreover, pCGS has been suggested to be cost-effective since using an analysis including ten different studies, only the use of pCGS is cost-effective, whilst other formulations (crystalline glucosamine sulfate, glucosamine sulfate, and glucosamine hydrochloride) are not [55]. However, since no Chinese work was included, the WG encourages specific research on economic costs in China.

Regarding AEs, hypothetical concerns have been raised regarding the safety of pCGS in patients having diabetes. Glucosamine is an amino sugar that might lead to hyperglycemia and insulin resistance, through the over-activation of the hexosamine pathway [66]. However, it was already known that when in plasma glucosamine “does not go back to glucose”, but is directly catabolized without any interference with glucose metabolism [67]. This statement is supported by the clinical trials’ data in human beings. At common doses used for OA treatment, pCGS showed no interference with glucose metabolism in subjects with normal plasma glucose levels and in most subjects with hyperglycemia, impaired insulin sensitivity, pre-diabetes or diabetes [52, 68]. In addition, a meta-analysis on the effects of glucosamine on glucose metabolism found that glucosamine, at the usual oral doses used in knee OA patients, is well-tolerated by normal, diabetic, or pre-diabetic patients [69]. In the PROOF trial a non-significant increase in glycated hemoglobin levels was found in overweight women who received pCGS during the follow-up period [70, 71]. Therefore, the WG recommends to advise caution at the start of treatment with glucosamine in diabetic patients [28].

#### Topical NSAIDs

In the background therapy of knee OA, topical non-steroidal anti-inflammatory drugs (NSAIDS) might be used as cyclic therapy if the patient is still symptomatic after treatment with SYSADOAs. Topical NSAIDs are considered safe [8], but their efficacy has been only demonstrated in short-term RCTs. Therefore, more data are needed for confirming their clinical impact [13]. The 2019 algorithm suggested that topical NSAIDs might be used in preference to oral NSAIDs, particularly in frail patients affected by knee OA, or prior to use of oral NSAIDs. The Chinese WG agreed with the use of topical NSAIDs for the control of persistent pain in knee OA.

### Step 2: advanced pharmacological treatment

Step 2 includes patients still suffering after the background therapy or with relevant limitations in the activities of daily living. In the second step, a relevant role is covered by oral NSAIDs. Based on the literature available with a particular attention to the safety of these medications [10], ESCEO makes a strong recommendation to the use of oral NSAIDs (selective or non-selective), but only intermittently and for a short period [13]. Furthermore, ESCEO underlines that the use of oral NSAIDs should be based on the patient risk profile, with a particular attention for cardiovascular, renal, and gastrointestinal profile [13]. Regarding the safety of oral NSAIDs, it is important to remember that, after removing rofecoxib, the cardiovascular risk decreased.

In the second part of step 2, intra-articular medications (hyaluronic acid and corticosteroids) are indicated. However, for both these intra-articular products, there is a weak evidence supporting the use of intra-articular medications in those who cannot take oral NSAIDs (e.g., allergy). A recent RCT, for example, found that physical therapy is better than corticosteroids injection for improving disability and pain in people with knee OA [72]. The reasons for this decision regarding intra-articular drugs are based on their efficacy, higher risk of AEs when compared to placebo and only having short-term RCTs supporting the use of these drugs [9, 13].

In conclusion, in step 2, the WG agreed to the judicious use of NSAIDs for acute exacerbation of knee OA, particularly in the case of an inflammatory component, after carefully considering the patient profile. The dose of NSAIDs should be the lowest effective dose.

### Step 3: last pharmacological treatment

Last pharmacological options for the still symptomatic patients are represented by short-term weak opioids. Tramadol may give benefits on analgesia in knee OA [73, 74], but a recent meta-analysis of the safety of oral opioids used in OA found an increased risk of gastrointestinal, central nervous system, and dermatological AEs when tramadol is compared with placebo [75]. For this reason, ESCEO gives only a weak recommendation to the use of short-term weak opioids, as the last pharmacological option before surgery [13]. A similar evidence base is available for duloxetine, for which a very limited efficacy is observed [13].

The WG concurs to the use of low-dose weak opioids, like tramadol, with the needed precaution for their known adverse events of nausea, somnolence, and vomiting.

### Step 4: end-stage disease management and surgery

Total knee replacement (TKR) is appropriate when all previous interventions have failed, in case of the patient still symptomatic, and when a significant loss in quality of life is present [76–78]. However, for patients in whom surgery is cotraindicated, the last pharmacological attempt could be oral or transdermal opioids [79], which should be prescribed following the guidelines for use of opioid analgesics in the management of non-cancer pain [80].

The WG adds that background physical therapy is to be continued for surgery-averse patients or those where surgery is contraindicated.

## Conclusions

Knee OA is a relevant problem in China, exponentially increasing, also for the rise of the risk factors associated with this condition, such as obesity. In this work, the WG has tried to summarize the reccomendations given in the 2019 ESCEO algorithm, highlighting specific areas where it applies to clinical practice Chinese people. In China, the steps of the 2019 algorithm are not only followed but also recognized as important. Our work further provides evidence-based and easy-to-follow advices regarding to establish a treatment algorithm in patients with knee OA, for practical implementations in the Chinese clinical practice.
